# Response of *cbbL* Carbon-Fixing Microorganisms to Flooding Depth During the Growing Season in Qinghai Lake Lakeshore Wetlands

**DOI:** 10.3390/biology15201815

**Published:** 2026-10-12

**Authors:** Hairui Zhao, Yanfen Yang, Chen Chen, Mingzhu Cao, Yuyu Ma, Shuchang Zhu, Xiong Xiong, Chenxi Wu, Kelong Chen

**Affiliations:** 1 School of Life Sciences, Qinghai Normal University, Xining 810008, China; 2 Key Laboratory of Natural Geography and Environmental Processes of Qinghai Province, Xining 810008, China; 3 Key Laboratory of Qinghai-Tibet Plateau Surface Process and Ecological Conservation, Ministry of Education, Qinghai Normal University, Xining 810008, China; 4 National Positioning Observation and Research Station of Qinghai Lake Wetland Ecosystem in Qinghai, National Forestry and Grassland Administration, Haibei 812300, China; 5 School of Geographical Sciences, Qinghai Normal University, Xining 810008, China; 6 State Key Laboratory of Freshwater Ecology and Biotechnology, Institute of Hydrobiology, Chinese Academy of Sciences, 7 Donghu South Road, Wuchang District, Wuhan 430072, China

**Keywords:** global climate change, Qinghai Lake, lakeshore wetland, *cbbL*, microbial carbon fixation

## Abstract

Lake-level rise driven by changing precipitation is increasing inundation in littoral wetlands, but responses of *cbbL*-harboring carbon-fixing microorganisms remain uncertain. A controlled soil-column experiment included four hydrological regimes: unflooded soil (CK), water level aligned with the soil surface (S0), and standing-water depths of 10 cm (S10) and 20 cm (S20). Amplicon sequencing of *cbbL* showed that the Shannon index was significantly higher in S20 than in CK (*p* = 0.0394), whereas Simpson, Chao1, and ACE did not differ significantly among treatments. Overall amplicon composition also lacked a significant treatment effect (PERMANOVA: R^2^ = 0.344, *p* = 0.112). *Pseudomonadota* represented 96.9–97.7% of taxonomically assigned sequences and remained dominant across treatments, although several genera varied in relative sequence proportion. Exploratory LEfSe screening identified *Rhodoblastus*-, *Hydrogenophaga*-, and *Azospirillum*-related lineages as candidate indicators of CK, S10, and S20, respectively. Multivariate soil profiles did not differ significantly among treatments (PERMANOVA: R^2^ = 0.374, *p* = 0.220). After collinearity filtering, EC had the highest normalized mean absolute out-of-fold SHAP contribution among EC, pH, and TC (43.8–59.1%). However, outer leave-one-out cross-validation produced negative predictive R^2^ values (−0.43 to −0.11), indicating limited model generalizability. Overall, S20 was associated with higher Shannon diversity than CK, while *Pseudomonadota* remained dominant and several genera varied in relative sequence proportion. EC should therefore be regarded as an exploratory candidate correlate of *cbbL* alpha diversity rather than a validated environmental driver, and this association requires further evaluation under natural hydrological conditions.

## Simple Summary

Rising lake levels can inundate lakeshore wetlands and affect microorganisms involved in carbon dioxide fixation. We maintained soil columns from a Qinghai Lake wetland under four water-level treatments and examined the microbial fraction detected using primers targeting the *cbbL* carbon-fixation gene. The Shannon index was significantly higher under the S20 treatment than under the non-inundated control, whereas overall amplicon community composition did not differ significantly among treatments. *Pseudomonadota* maintained the highest relative sequence proportion across all water levels, while the relative proportions of several genera varied among treatments. In the XGBoost models, electrical conductivity had the highest normalized mean absolute out-of-fold SHAP contribution among the three retained soil variables, although predictive performance for held-out samples was limited. These findings reveal modest changes in diversity and relative taxonomic composition associated with deeper inundation and provide testable directions for larger-scale field studies.

## 1. Introduction

Climate-driven shifts in precipitation, evapotranspiration, and runoff are reorganizing wetland hydrology and carbon cycling [1,2,3]. On the Qinghai–Tibet Plateau, recent warming and wetting have altered the storage and extent of many lakes [2,3]. As the largest inland saline lake on the Qinghai–Tibet Plateau, Qinghai Lake has experienced a sustained increase in water level since 2004, mainly in response to greater precipitation and river inflow [4,5,6]. The resulting shoreline advance exposes littoral soils to contrasting inundation conditions that may modify wetland carbon processes.

Wetland soils are globally important reservoirs of organic carbon [7], and carbon storage in alpine wetlands depends on hydrothermal and edaphic conditions [3,8]. Microbial autotrophy provides a pathway of soil CO_2_ incorporation in addition to plant photosynthesis [9]. The Calvin–Benson–Bassham cycle is a major microbial carbon-fixation pathway, and the *cbbL* gene, which encodes the large subunit of form I RubisCO, is commonly used to survey microorganisms with this metabolic potential [9,10,11,12]. Around Qinghai Lake, *cbbL* amplicon assemblages differ among wetland types and covary with soil moisture, temperature, total carbon, and total nitrogen [13].

Inundation can modify soil aeration, redox conditions, and exchanges between lake water and soil, thereby affecting EC, pH, and carbon–nitrogen pools [14,15]. Carbon fractions and microbial biomass carbon have been associated with wetland carbon-fixing communities [16], while moisture manipulations have linked variation in *cbbL* assemblages to soil carbon, nitrogen, and pH [17,18]. These observations suggest that soil ionic conditions and measured carbon–nitrogen stocks may provide complementary information about *cbbL* diversity. However, their relative associations across controlled lake-water inundation depths remain unresolved.

Previous studies around Qinghai Lake have mainly compared different wetland types or examined responses to simulated precipitation and warming [13,18,19]. Controlled evidence concerning the effects of lake-water inundation depth remains limited. We therefore established CK, S0, S10, and S20 treatments to characterize the alpha diversity and relative taxonomic composition of detectable *cbbL* amplicons and to examine their statistical associations with measured soil properties. We hypothesized that (i) the transition from non-inundated to inundated conditions and increasing water depth would alter detectable *cbbL* sequence diversity and relative composition; and (ii) variation in *cbbL* alpha diversity would be associated with soil EC, pH, and carbon–nitrogen stocks. These hypotheses concern the detectable *cbbL* amplicon pool and do not imply changes in absolute microbial abundance or actual carbon-fixation rates.

## 2. Materials and Methods

### 2.1. Study Area

**Figure 1:**
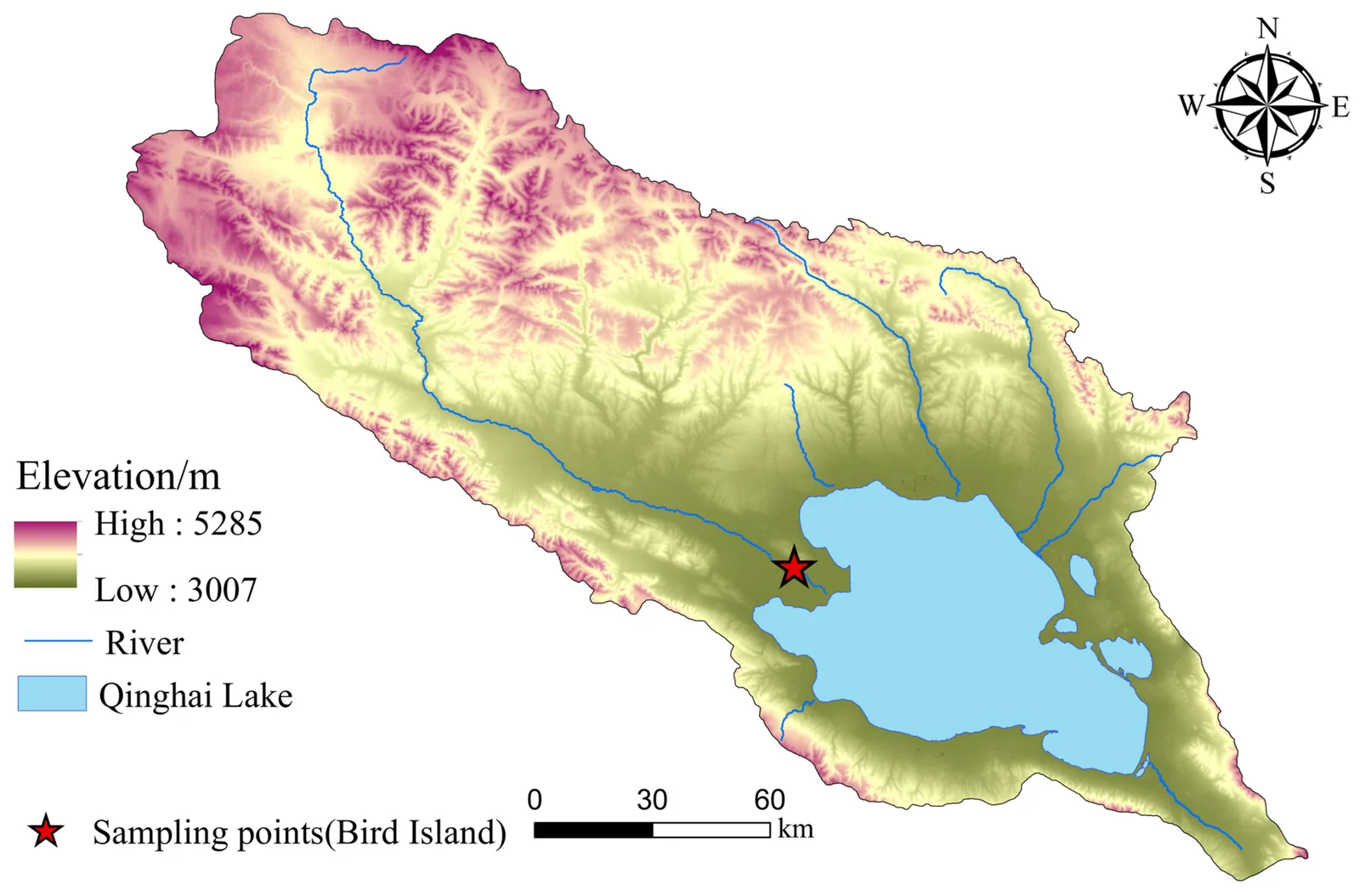
Overview of the study area.

Bird Island Station, located along the northwestern margin of Qinghai Lake, was selected as the experimental site (36°57′–37°04′ N, 99°44′–99°54′ E; elevation, 3194–3220 m; Figure 1). This alpine littoral wetland has a semiarid climate, with an annual mean air temperature of −0.7 °C and annual precipitation of approximately 420 mm. Plant cover exceeds 60%, forming a 30–40 cm canopy dominated by *Leymus secalinus*, *Artemisia frigida*, and *Poa alpigena*. Under the World Reference Base system, the soil is classified as Kastanozem. Qinghai Lake has undergone sustained water-level rise since 2004. Observations from 2014 to 2018 indicate that the inundated wetland expanded by 21.86 km^2^ and that the inundation front advanced locally by 20–500 m [20].

### 2.2. Soil-Column Establishment, Inundation Treatments, Sample Collection, and Soil Physicochemical Analyses

Field sampling was conducted at 12 independent locations in August 2024, with a minimum distance of 1 m between adjacent locations. A corer measuring 25 cm in diameter and 50 cm in height was used to recover the 0–35 cm soil profile from each location. The profiles were handled separately and repacked in their original vertical sequence into individual acrylic cylinders with an inner diameter of 24 cm, an outer diameter of 25 cm, and a height of 50 cm. Material collected from different locations was not combined. The base of each cylinder was sealed, and 1.6 cm lateral openings were positioned at 10 cm intervals. An opaque covering around the sidewall prevented light penetration and maintained dark subsurface conditions.

Random allocation placed three columns in each of four hydrological treatments. CK remained unflooded, S0 was maintained with the water level aligned with the soil surface, and S10 and S20 received standing-water depths of 10 and 20 cm, respectively. Individual columns represent independent experimental units. Water used to maintain the inundation treatments was collected locally from Qinghai Lake. These four treatments were selected from the broader soil-column platform reported previously [21], whereas the present study specifically examines *cbbL* amplicon data. Following their establishment in August 2024, the soil columns were maintained under their respective water-level treatments for six months to allow stabilization. The same water levels were then maintained until soil sampling in July 2025. At mid-growing season, one 0–10 cm soil sample was collected from each column, yielding 12 independent biological samples (*n* = 3 per treatment), with no temporal or within-column depth replication. Samples were sieved through a 2 mm mesh, transported on ice, and divided into subsamples stored at −4 °C for physicochemical analyses and −80 °C for molecular analysis. Soil pH and EC were measured using a PHS-25 pH meter and a DDS-307 conductivity meter, respectively (Shanghai Instruments and Electrical Science Co., Ltd., Shanghai, China). TC and TN were quantified using an ECS4024 elemental analyzer (LICA United Technology Co., Ltd., Beijing, China). TOC was determined by potassium dichromate oxidation with external heating, TIC was calculated as TC − TOC, and MBC was measured by chloroform fumigation–K_2_SO_4_ extraction [21].

### 2.3. DNA Extraction, PCR Amplification, and Sequence Processing

DNA was recovered from each soil sample with the PowerSoil DNA Isolation Kit (MoBio Laboratories, Carlsbad, CA, USA), and its integrity was evaluated on a 1% agarose gel. Primers K2f (5′-ACCAYCAAGCCSAAGCTSGG-3′) and V2r (5′-GCCTTCSAGCTTGCCSACCRC-3′) targeted an approximately 490 bp fragment of *cbbL* [22]. Following purification, the amplicons underwent 2 × 300 bp paired-end sequencing on an Illumina NextSeq 2000 platform. Bioinformatic processing was conducted in QIIME 2 (release 2022.11). Cutadapt excised barcode and primer regions, after which VSEARCH joined the paired reads, screened sequence quality, and performed dereplication. Potential chimeras were discarded with the de novo UCHIME procedure before clustering. FrameBot correction was applied to potential frameshift errors, and sequences that could not be corrected, together with singleton sequences, were removed. The retained sequences were grouped de novo in VSEARCH at 97% identity, and the centroid of each cluster served as its representative sequence. This cutoff was retained to facilitate comparison with previous *cbbL* amplicon studies and to limit the influence of residual sequencing errors. Accordingly, OTUs were interpreted as sequence-based operational units rather than microbial species. Representative OTUs were searched against the NCBI nucleotide collection (nt) using BLASTn (NCBI BLAST+ v2.16.0). The best-supported sequence matches and their associated NCBI Taxonomy records guided taxonomic assignment. Subsequent community analyses used the quality-filtered OTU abundance matrix. Raw reads are deposited in the NCBI Sequence Read Archive under BioProject PRJNA1520236.

### 2.4. Statistical Analysis

All analyses were performed in R version 4.4.2, with each soil column treated as an independent biological replicate (*n* = 3 per treatment). Rarefaction curves and observed OTU counts described the accumulation of *cbbL* sequence units. Shannon, Simpson, Chao1, and ACE indices were calculated, and treatment differences were tested using one-way ANOVA followed by Tukey’s HSD test.

Bray–Curtis dissimilarities were analyzed by principal coordinate analysis (PCoA), and overall compositional differences were tested using PERMANOVA. Relative sequence proportions were summarized at the phylum and genus levels. LEfSe analysis used *p* < 0.05 and an LDA score > 3.0. Given the three replicates per treatment, LEfSe results were interpreted as exploratory candidate taxa.

Standardized environmental variables were analyzed by PCA and Pearson correlation analysis. To reduce multicollinearity, TC was selected to represent the highly correlated TC, TN, TOC, TIC, and MBC variables. EC, pH, and TC were included in XGBoost models of the four alpha-diversity indices. The learning rate, maximum tree depth, sample-subsampling ratio, and feature-subsampling ratio were set to 0.10, 3, 0.80, and 0.80, respectively. A maximum of 100 boosting rounds was allowed, with the optimal number selected by inner five-fold cross-validation and early stopping after 10 rounds without improvement. Outer leave-one-out cross-validation was used to calculate held-out SHAP values, cross-validated R^2^, and RMSE. LOESS curves described EC–SHAP relationships without inferring ecological thresholds.

## 3. Results

### 3.1. Response of cbbL-Harboring Microbial Community Diversity to Inundation

**Figure 2:**
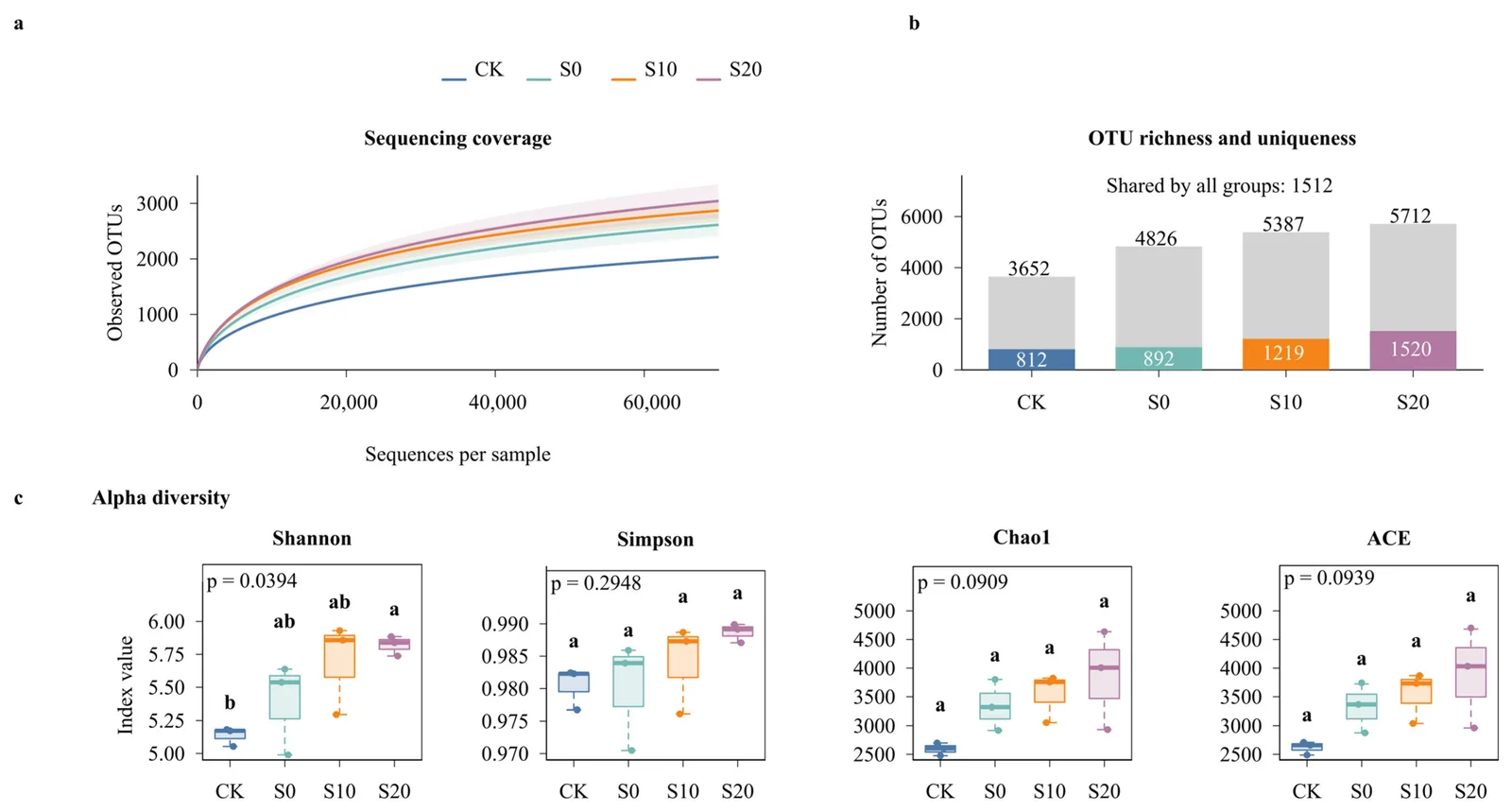
Sequencing effort, pooled OTU sets, and alpha diversity of *cbbL* amplicons. (**a**) Rarefaction curves with shaded uncertainty bands. (**b**) Numbers of OTUs detected in the pooled three-sample set for each treatment and numbers observed only in the corresponding pooled set; 1512 OTUs were shared across all four treatments. (**c**) Shannon, Simpson, Chao1, and ACE indices. Each point represents an independent biological sample (*n* = 3 per treatment), and boxplots summarize sample distributions. Reported *p* values are from overall one-way ANOVA tests. Different lowercase letters indicate significant pairwise differences according to Tukey’s HSD test (*p* < 0.05).

The rarefaction curves, OTU composition, and alpha diversity of *cbbL* amplicon sequences under different inundation treatments are shown in Figure 2. As the number of sequences increased, the observed OTU count initially increased rapidly, followed by a gradual decline in the rate of new OTU detection. When the sequencing effort approached 70,000 sequences, the curves gradually flattened (Figure 2a). These rarefaction curves represent only the accumulation of *cbbL* OTUs detectable with the primer pair used and do not indicate complete coverage of all carbon-fixing microorganisms. At the same sequencing effort, the observed OTU counts generally followed the order S20 > S10 > S0 > CK, indicating an increasing trend in the number of detectable *cbbL* sequence units with increasing inundation depth.

The pooled OTU sets of CK, S0, S10, and S20 contained 3652, 4826, 5387, and 5712 OTUs, respectively (Figure 2b), corresponding to values 32.1%, 47.5%, and 56.4% higher in the three inundation treatments than in CK. These values represent the union of OTUs detected across the three independent samples within each treatment and were used only to describe the observed treatment-level OTU sets; they do not represent the richness of individual samples. A total of 1512 OTUs were shared among all four treatments. The numbers of OTUs observed exclusively within the pooled sets of CK, S0, S10, and S20 were 812, 892, 1219, and 1520, respectively, with the value for S20 being 87.2% higher than that for CK. These results describe differences among the pooled OTU sets observed in the current sequencing dataset.

Alpha-diversity analysis showed a significant difference in the Shannon index among treatments (*p* = 0.0394; Figure 2c). The Shannon index was significantly higher in S20 than in CK, whereas S0 and S10 showed intermediate values. The Simpson index generally increased with inundation depth, but the overall difference was not significant (*p* = 0.2948). Chao1 and ACE also showed increasing trends, although neither differed significantly among treatments (*p* = 0.0909 and *p* = 0.0939, respectively). Thus, S20 was primarily characterized by a higher Shannon index, whereas the richness estimators did not differ significantly among treatments. This contrasting response suggests that the observed diversity difference may be associated more closely with the relative-abundance distribution or evenness of OTUs than with a significant increase in OTU richness.

### 3.2. Effects of Inundation on the Taxonomic Composition and Indicator Taxa of cbbL-Harboring Microbial Communities

**Figure 3:**
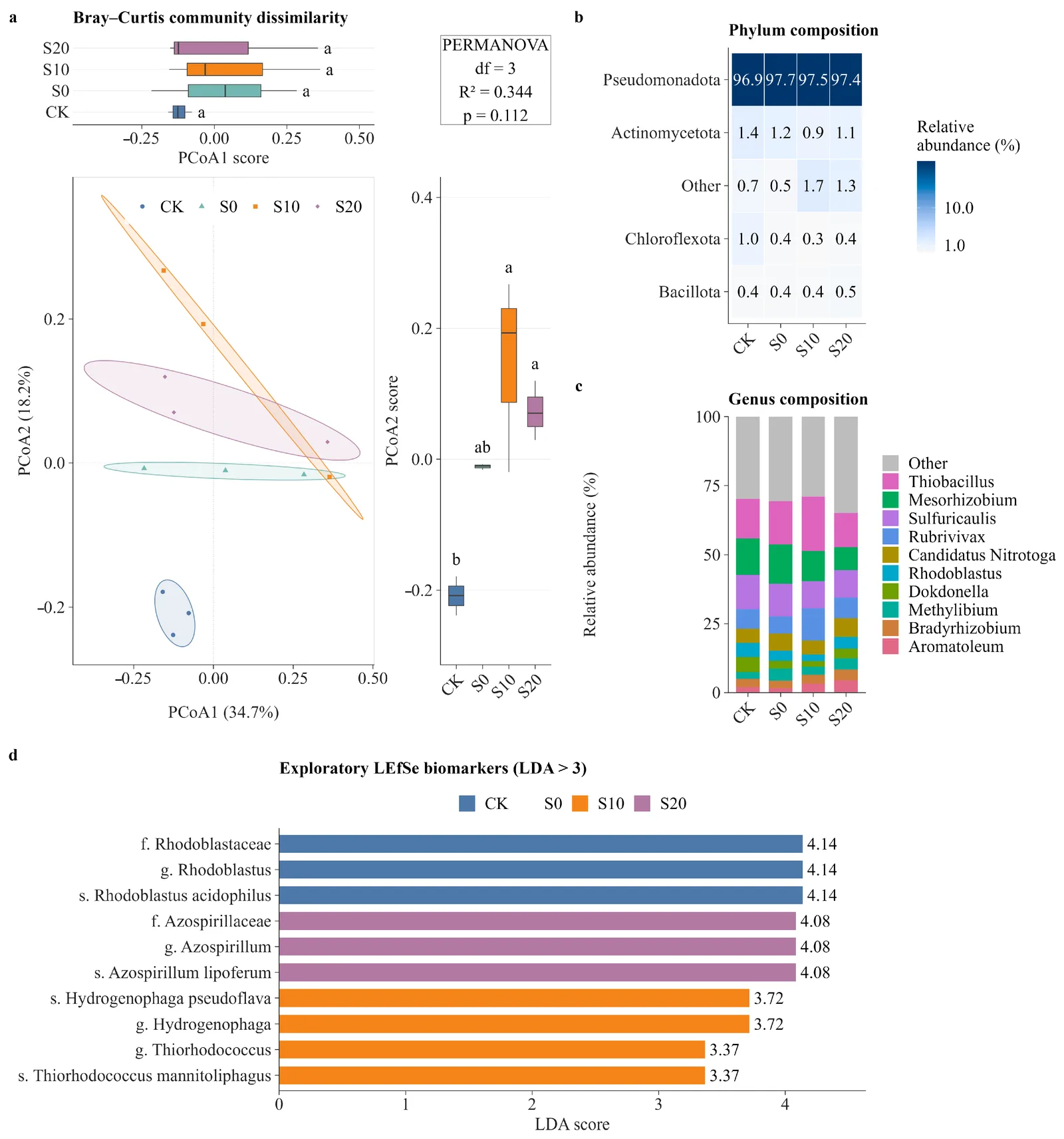
Community composition and differentially abundant taxa of *cbbL*-harboring microbial communities under different inundation treatments. (**a**) Principal coordinate analysis (PCoA) based on Bray–Curtis dissimilarities. Different lowercase letters indicate significant differences among treatments (*p* < 0.05). (**b**) Relative abundance of microbial taxa at the phylum level. (**c**) Relative abundance at the genus level. (**d**) Linear discriminant analysis effect size (LEfSe) bar plot showing differentially abundant taxa among treatments with linear discriminant analysis (LDA) scores > 3. Colored shaded ellipses in panel (**a**) summarize within-treatment dispersion in ordination space and are shown for visualization. In panel (**d**), the prefixes f., g., and s. denote family, genus, and species, respectively.

PCoA based on Bray–Curtis distances showed differences in sample positions among the inundation treatments (Figure 3a). PCoA1 and PCoA2 explained 34.7% and 18.2% of the community variation, respectively, accounting for 52.9% of the total variation. CK samples were mainly located on the negative side of PCoA2, whereas S10 and S20 samples were primarily positioned on the positive side, with S0 samples occupying an intermediate position. PCoA1 scores did not differ significantly among treatments. The PCoA2 scores were significantly higher in S10 and S20 than in CK, whereas S0 showed intermediate values. PERMANOVA did not detect a significant overall difference in *cbbL* amplicon community composition among treatments (R^2^ = 0.344, *p* = 0.112).

At the phylum level, Pseudomonadota accounted for 96.9–97.7% of the taxonomically assigned *cbbL* amplicon sequences and was dominant across all treatments (Figure 3b). Actinomycetota, Chloroflexota, and Bacillota occurred at relatively low abundances. The relative abundances of Chloroflexota were 1.0%, 0.4%, 0.3%, and 0.4% in CK, S0, S10, and S20, respectively. These relative abundances were calculated from *cbbL*-targeted amplicon sequences and represent the composition of the *cbbL*-harboring community.

At the genus level, the relative sequence proportions of several taxonomic groups varied among treatments (Figure 3c). The mean relative abundances of *Thiobacillus* were 14.2%, 15.8%, 19.6%, and 12.4% in CK, S0, S10, and S20, respectively. Its highest proportion.

Occurred in S10, whereas its proportion in S20 was lower than that in S10. The corresponding relative abundances of *Mesorhizobium* were 13.2%, 14.2%, 11.0%, and 8.3%, with the lowest value observed in S20. The “Other” category, representing the combined relative abundance of genera not displayed separately, accounted for 29.8%, 30.6%, 29.0%, and 34.9%, respectively. Overall, Pseudomonadota remained dominant across treatments, whereas several genera varied in their relative sequence proportions. These proportional variations did not correspond to a significant overall difference in community composition.

The exploratory LEfSe analysis identified 10 taxonomic entries with LDA scores greater than 3 (Figure 3d). Candidate indicator taxa in CK were mainly affiliated with the *Rhodoblastus* lineage (LDA = 4.14), whereas those in S20 were primarily affiliated with the *Azospirillum* lineage (LDA = 4.08). Candidate indicator taxa in S10 included *Hydrogenophaga*, *H. pseudoflava* (LDA = 3.72), *Thiorhodococcus*, and *T. mannitoliphagus* (LDA = 3.37). No taxonomic entries with LDA scores greater than 3 were detected in S0. Given that each treatment included only three biological replicates, the LEfSe results were used to identify candidate indicator taxa rather than to validate treatment-specific biomarkers.

### 3.3. Soil Environmental Variation and Its Associations with the Alpha Diversity of cbbL-Harboring Communities

**Figure 4:**
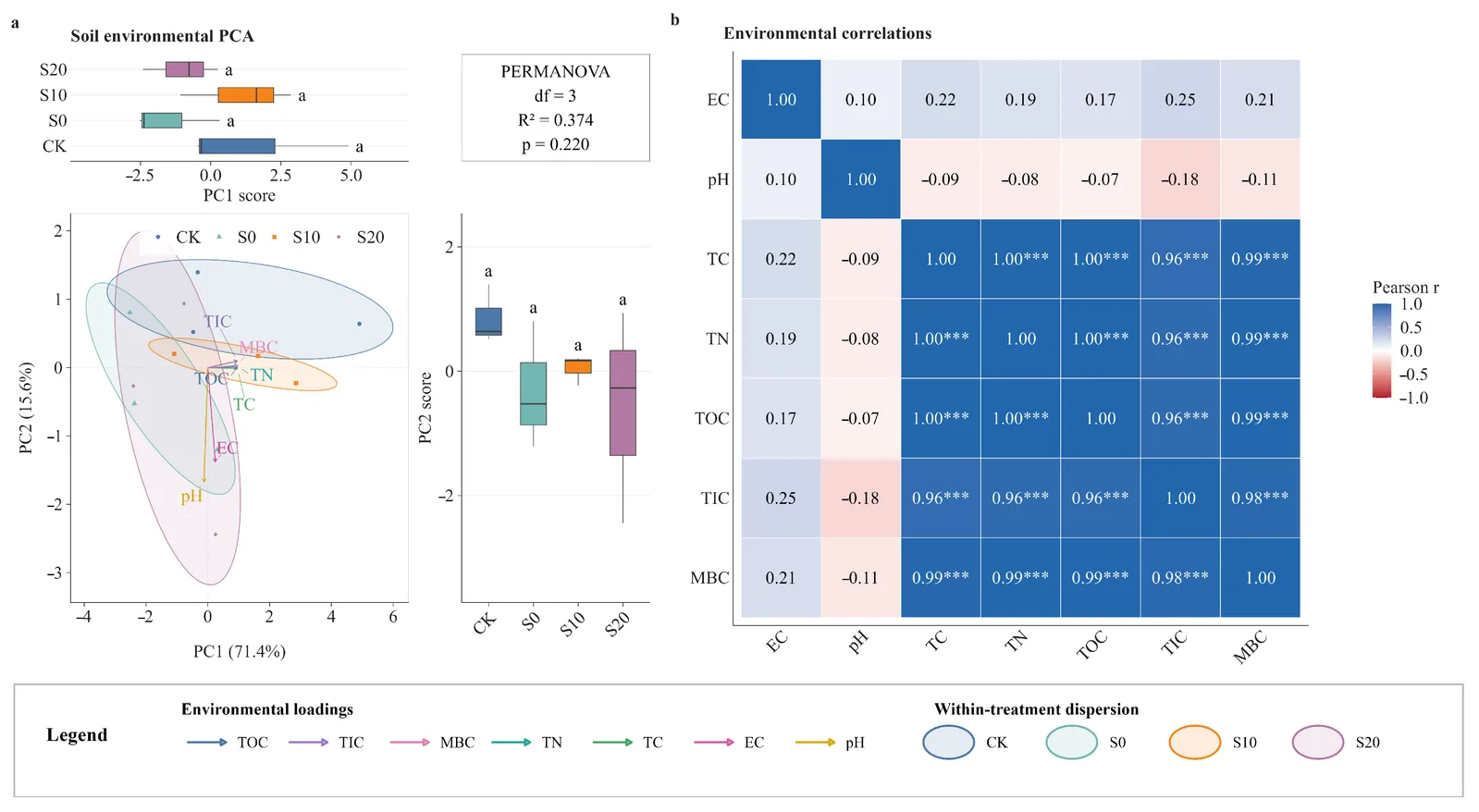
Relationships among soil environmental variables and their predictive contributions to alpha-diversity. (**a**) Principal component analysis (PCA) of soil environmental variables. Identical lowercase letters indicate no significant differences according to Tukey’s multiple-comparison test (*p* > 0.05). (**b**) Pearson correlations among environmental variables; *** indicates *p* < 0.001. In panel (**a**), arrow colors identify environmental-variable loadings (TOC, TIC, MBC, TN, TC, EC, and pH), whereas ellipse colors identify CK, S0, S10, and S20; the shaded ellipses summarize within-treatment dispersion in ordination space.

Principal component analysis of the soil environmental variables showed that PC1 and PC2 explained 71.4% and 15.6% of the total variance, respectively, accounting for a cumulative 87.0% (Figure 4a). However, several variables included in the PCA were highly correlated, and TIC was calculated as the difference between TC and TOC. Therefore, the high cumulative variance explained by the first two axes partly reflected covariation and structural redundancy among the variables rather than independent environmental gradients. S10 samples were mainly distributed along the positive direction of PC1, whereas S0 samples tended to occur along its negative direction. CK and S20 exhibited relatively high within-treatment dispersion. The four treatments did not form an ordered CK–S0–S10–S20 sequence, and their distributions overlapped substantially. Consistent with this overlap, PERMANOVA detected no significant overall difference in the multivariate soil environment among treatments (R^2^ = 0.374, *p* = 0.220).

Pearson correlation analysis showed that the correlation coefficients among TC, TN, and TOC approached 1.00. These variables were also strongly and positively correlated with TIC and MBC (r = 0.96–0.99, *p* < 0.001; Figure 4b). In contrast, EC was weakly and positively correlated with the other environmental variables (r = 0.10–0.25), whereas pH was weakly and negatively correlated with the carbon- and nitrogen-related variables (r = −0.07 to −0.18). These relationships indicate substantial covariation and information overlap but do not imply that the variables are functionally equivalent or that they all represent nutrient availability. TC, TOC, and TIC represent distinct but structurally related soil carbon pools, TN represents the total nitrogen stock, and MBC reflects microbial biomass. To reduce multicollinearity in the subsequent analyses, TC was selected to represent this highly correlated variable set, and only EC, pH, and TC were retained.

**Figure 5:**
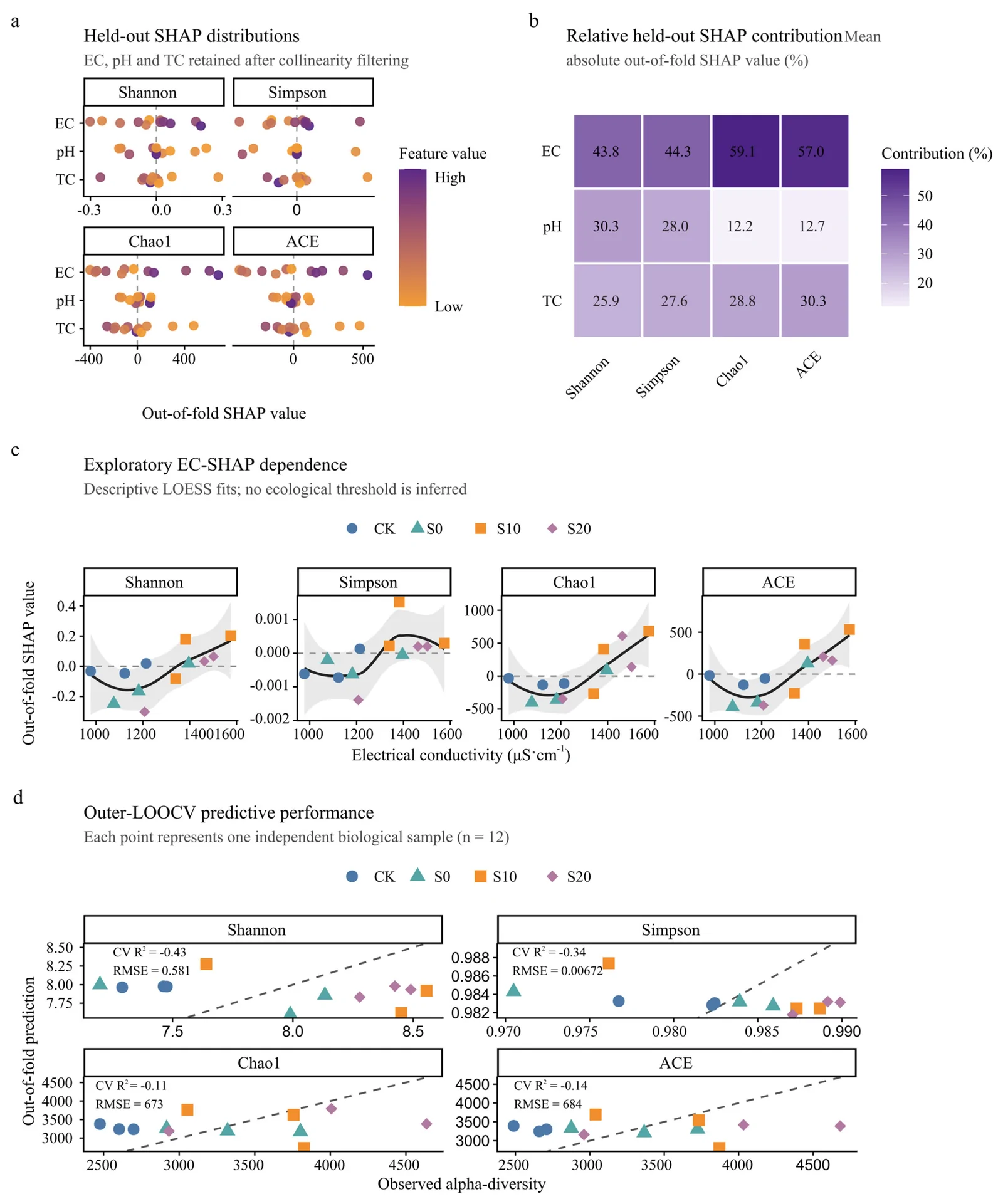
Exploratory XGBoost–SHAP analysis of the relationships between environmental factors and the α-diversity of *cbbL*-harboring carbon-fixing microorganisms. (**a**) Distributions of held-out SHAP values for EC, pH, and TC retained after collinearity filtering across the Shannon, Simpson, Chao1, and ACE indices. Point colors represent the values of the corresponding environmental variables. (**b**) Relative contributions of environmental variables calculated from the mean absolute held-out SHAP values. (**c**) Dependence relationships between EC and held-out SHAP values. (**d**) Relationships between observed and predicted values obtained using outer leave-one-out cross-validation. Dashed lines represent the 1:1 reference lines; the cross-validated coefficient of determination (CV R^2^) and root mean square error (RMSE) are shown. In panel (**c**), black curves are descriptive LOESS fits, and grey shading represents 95% pointwise confidence bands.

To investigate the statistical associations between soil properties and alpha diversity, SHAP analysis based on outer leave-one-out cross-validation was used to evaluate the relative contributions of EC, pH, and TC to the prediction of the Shannon, Simpson, Chao1, and ACE indices. Among the three retained variables, EC had the highest normalized mean absolute out-of-fold SHAP contribution for all four indices (Figure 5a,b). Its relative contributions to Shannon, Simpson, Chao1, and ACE were 43.8%, 44.3%, 59.1%, and 57.0%, respectively. The corresponding contributions of TC were 25.9%, 27.6%, 28.8%, and 30.3%, whereas those of pH were 30.3%, 28.0%, 12.2%, and 12.7%. EC contributed relatively more to the Chao1 and ACE models, whereas pH contributed relatively more to the Shannon and Simpson models.

The EC–SHAP dependence plots showed apparent nonlinear variation (Figure 5c). At lower EC values, the SHAP values were generally negative and shifted toward positive values as EC increased. This pattern was more evident for Shannon, Chao1, and ACE. However, the observations were widely dispersed. The LOESS curves were therefore used only to summarize patterns within the present dataset and not to identify ecological thresholds.

Outer leave-one-out cross-validation yielded predictive R^2^ values of −0.43, −0.34, −0.11, and −0.14 for the Shannon, Simpson, Chao1, and ACE models, respectively. The corresponding RMSE values were 0.581, 0.00672, 673, and 684 (Figure 5d). The negative cross-validated R^2^ values indicate limited predictive performance for independently held-out samples. Within these exploratory models, EC had the highest normalized mean absolute out-of-fold SHAP contribution, particularly in the Chao1 and ACE models. However, given the small sample size and limited predictive performance, this ranking does not provide evidence that EC is an independent environmental driver. EC should therefore be regarded only as a candidate soil property associated with alpha diversity.

## 4. Discussion

### 4.1. Responses of cbbL-Harboring Microbial Communities to Changes in Inundation

The number of detectable *cbbL* OTUs in the pooled treatment datasets generally increased with inundation depth. The Shannon index was significantly higher in S20 than in CK, whereas the Simpson, Chao1, and ACE indices showed only nonsignificant upward trends. This pattern suggests that the response to deeper inundation may have been reflected primarily in changes in the diversity and relative abundance distribution of *cbbL* sequence variants rather than in a significant increase in richness. Previous studies have identified water availability as an important factor influencing soil *cbbL* functional communities [17,23], potentially through its effects on material transport, oxygen diffusion, and soil environmental conditions [24,25,26].

Microbial diversity does not respond consistently to inundation in natural wetlands. Studies in the Poyang Lake wetland found that community composition varied with water level and inundation duration, whereas diversity showed no consistent trend [27]. Bacterial alpha diversity in a lakeshore wetland exhibited a unimodal pattern along an elevation gradient and was lower in the flooding year [28]. In naturally inundated areas around Qinghai Lake, microbial alpha diversity declined following inundation, whereas beta diversity responded nonlinearly to inundation duration [29]. These contrasting patterns indicate that community responses may be jointly influenced by the target community, inundation duration, water-level fluctuations, vegetation, and other factors.

PCoA showed differences in sample positions among treatments, but PERMANOVA detected no significant overall difference in community composition (R^2^ = 0.344, *p* = 0.112), indicating only limited variation in *cbbL* amplicon composition among inundation treatments. Compared with the significant changes reported along natural water-level gradients [27,28,29], this difference may reflect variation in the target communities and hydrological conditions examined.

At the phylum level, *Pseudomonadota* accounted for more than 96% of the taxonomically assigned *cbbL* amplicon sequences and remained dominant across treatments, consistent with findings from croplands, grasslands, and karst wetlands [16,30]. Its metabolic diversity may contribute to this predominance [31]. The relative proportion of *Chloroflexota* decreased under inundation, but this pattern requires cautious interpretation because of its low abundance and the absence of statistical support.

At the genus level, the relative sequence proportions of several taxa varied among treatments. Candidate indicator taxa in S10 included *Thiobacillus*, *Hydrogenophaga*, and *Thiorhodococcus*, some members of which have the potential for sulfur-compound oxidation, hydrogen or carbon monoxide oxidation, and photoautotrophy [32,33,34,35]. *Azospirillum*- and *Rhodoblastus*-related lineages were identified as candidate indicator taxa in S20 and CK, respectively [9,35]. However, the LEfSe results represent only exploratory differences in relative sequence proportions. Overall, *Pseudomonadota* remained dominant, whereas Shannon diversity and the relative proportions of several lower-level taxa varied among inundation treatments [23,36].

### 4.2. Associations Between Soil Properties and the Alpha Diversity of cbbL Amplicon Assemblages

The first two axes of the soil environmental PCA explained 87.0% of the total variance. This high proportion largely reflected the strong covariance and structural redundancy among TC, TN, TOC, TIC, and MBC, particularly because TIC was calculated as the difference between TC and TOC, rather than multiple independent environmental gradients. S10 and S0 samples tended to occur along the positive and negative directions of PC1, respectively, whereas CK and S20 showed greater within-group dispersion. However, the treatments did not form an ordered sequence and overlapped substantially. PERMANOVA likewise detected no significant overall treatment effect on the soil environment (R^2^ = 0.374, *p* = 0.220). Wetland hydrological processes can affect soil aeration, ion transport, organic matter decomposition, and nutrient transformation [27,37], but the present results provide no statistical evidence of significant overall environmental differentiation among treatments.

TC, TN, TOC, TIC, and MBC covaried strongly but represented distinct soil properties. TC, TOC, and TIC describe structurally related carbon pools, TN represents total nitrogen content, and MBC reflects microbial biomass carbon [16,38]. Including these collinear variables simultaneously may destabilize variable-importance rankings [39]. Therefore, TC was selected to represent this correlated variable set and was modeled with EC and pH in the subsequent XGBoost analyses.

After collinearity filtering, EC had the highest normalized mean absolute held-out SHAP contribution among the three retained variables (43.8–59.1%), with greater contributions to Chao1 and ACE than to Shannon and Simpson. Its weak Pearson correlations with the other variables do not conflict with the SHAP results because Pearson correlation describes linear relationships, whereas SHAP reflects predictive information within a nonlinear multivariable model [40]. EC represents the overall soluble-ion level of the soil solution, and studies of saline soils and wetlands have associated EC and salinity with microbial community composition [41,42]. TC contributed 25.9–30.3%, providing complementary information on the soil carbon pool [16], whereas the relatively greater contribution of pH to Shannon and Simpson may be related to microbial physiology and soil chemical conditions [43].

Qinghai Lake is a high-altitude inland saline lake, and the inundation treatments used lake water collected from the study site. EC therefore provides an integrated indicator of soil-solution ionic conditions. Previous studies have associated EC and salinity with microbial community variation in Qinghai Lake wetlands [44] and other lakes on the Qinghai–Tibetan Plateau [45,46,47]. The relatively high SHAP contribution of EC may reflect ionic information not represented by the correlated carbon- and nitrogen-related variables, but it does not establish a stronger ecological effect than that of soil carbon and nitrogen stocks. Because outer leave-one-out cross-validation produced negative predictive R^2^ values (−0.43 to −0.11), model generalizability was limited. EC should therefore be regarded only as a candidate soil property associated with the alpha diversity of *cbbL*-harboring carbon-fixing microorganisms, without implying an ecological threshold or causal relationship.

### 4.3. Study Limitations

The soil-column experiment enabled controlled comparisons across inundation depths but could not fully reproduce natural water-level fluctuations or hydrological connectivity. The absence of plants excluded rhizosphere inputs and plant–microbe interactions. Because the microbial community in the lake water used for inundation was not characterized, waterborne immigration could not be distinguished from changes in the indigenous soil community. The six-month stabilization period may have been insufficient for the repacked soil to recover fully. Moreover, the three biological replicates per treatment limited statistical power and model generalizability. Therefore, the observed community patterns and environmental associations should be considered exploratory. Future studies should combine long-term in situ monitoring, paired lake-water and soil analyses, plant-inclusive experiments, and larger sample sizes to validate these findings.

## 5. Conclusions

This study used a controlled inundation experiment to compare *cbbL* amplicon sequence profiles under different hydrological treatments in a Qinghai Lake lakeshore wetland. The Shannon index was significantly higher in S20 than in CK, whereas the Simpson, Chao1, and ACE indices did not differ significantly among treatments. This pattern indicates that the response of detected *cbbL* sequence variants to inundation was mainly reflected in the diversity and evenness components captured by the Shannon index.

Pseudomonadota accounted for 96.9–97.7% of the taxonomically classified *cbbL* amplicon sequences and maintained a high relative sequence proportion across all treatments, indicating broadly stable composition at the phylum level. PERMANOVA detected no significant overall differences among treatments in either *cbbL* amplicon community composition or multivariate soil environmental profiles. The observed genus- and species-level differences and the LEfSe results therefore provide candidate lineages for further investigation of hydrological responses.

After collinearity filtering, EC had the highest normalized mean absolute out-of-fold SHAP contribution in the revised XGBoost models, although outer leave-one-out cross-validation indicated limited predictive performance. Collectively, these analyses identified EC as a candidate soil property associated with the alpha diversity of detected *cbbL* sequences that warrants further investigation. Future studies incorporating larger sample sizes, long-term in situ observations, and measurements of absolute abundance, gene expression, and actual carbon-fixation rates could further clarify the responses of lakeshore wetland *cbbL* functional groups to hydrological variation.

## Data Availability

The data presented in this study are available on request from the Corresponding author due to privacy or ethical restrictions.
